# Association between Directly Observed Therapy and Treatment Outcomes in Multidrug-Resistant Tuberculosis: A Systematic Review and Meta-Analysis

**DOI:** 10.1371/journal.pone.0150511

**Published:** 2016-03-01

**Authors:** Jia Yin, Jinqiu Yuan, Yanhong Hu, Xiaolin Wei

**Affiliations:** 1 The Jockey Club School of Public Health and Primary Care, The Chinese University of Hong Kong, Shatin, Hong Kong; 2 Dalla Lana School of Public Health, University of Toronto, Toronto, Ontario, Canada; Fundació Institut d’Investigació en Ciències de la Salut Germans Trias i Pujol, Universitat Autònoma de Barcelona, SPAIN

## Abstract

**Background:**

Multidrug-resistant tuberculosis (MDR-TB) represents a major obstacle towards successful TB control. Directly observed therapy (DOT) was recommended by WHO to improve adherence and treatment outcomes of MDR-TB patients, however, the effectiveness of DOT on treatment outcomes of MDR-TB patients was mixed in previous studies. We conducted this systematic review and meta-analysis to assess the association between DOT and treatment outcomes and to examine the impact of different DOT providers and DOT locations on successful treatment outcomes in MDR-TB patients.

**Methods:**

We searched studies published in English between January 1970 and December 2015 in major electronic databases. Two reviewers independently screened articles and extracted information of DOT, treatment success rate and other characteristics of studies. Random effects model was used to calculate the pooled treatment success rate and 95% confidence interval (CI). Sub-group analyses were conducted to access factors associated with successful treatment outcomes.

**Results:**

A total of 31 articles 7,466 participants were included. Studies reporting full DOT (67.4%, 95% CI: 61.4–72.8%) had significantly higher pooled treatment success rates than those reporting self-administration therapy (46.9%, 95% CI: 41.4–52.4%). No statistically difference was found among DOT provided by healthcare providers (65.8%, 95% CI: 55.7–74.7%), family members (72.0%, 95% CI: 31.5–93.5%) and private DOT providers (69.5%, 95% CI: 57.0–79.7%); and neither did we find significantly difference on pooled treatment success rates between patients having health facility based DOT (70.5%, 95% CI: 61.5–78.1%) and home-based DOT (68.4%, 95% CI: 51.5–81.5%).

**Conclusion:**

Providing DOT for a full course of treatment associated with a higher treatment success rate in MDR-TB patients.

## Introduction

Multidrug-resistant tuberculosis (MDR-TB) was defined as strains of Mycobacterium tuberculosis resistant to at least isoniazid and rifampicin, i.e., the two first-line anti-TB drugs [1]. The slow response of public health agencies to MDR-TB has led to a rapid increase of MDR-TB epidemic worldwide [2]. In 2013, MDR-TB accounted for 210,000 deaths, or 14% of the global TB mortalities, which has been regarded as the major obstacle to achieve the Millennium Development Goals for TB [2]. According to World Health Organization’s (WHO) guideline on drug-resistance TB management, MDR-TB treatment regimen takes at least 20 months in two treatment phases: the intensive phase and the continuation phase [3]. For a standardized regimen, WHO suggested to use four or more effective second-line anti-TB drugs (including one injectable and three oral drugs) plus pyrazinamide in the eight-month intensive phase, and all the oral in the continuation phase which normally lasts for 12–18 months [3]. Treating MDR-TB patients with second-line anti-TB drugs is more toxic, less effective and much more expensive compared with treating drug-susceptible TB patients with first-line anti-TB drugs. In addition, the prolonged treatment regimens increase the risk of patients’ non-adherence to the medicine, and result in unsuccessful treatment outcomes [4, 5].

Improving treatment adherence has been the core values of the Directly Observed Treatment, Short Course (DOTS) strategy for TB control. WHO has raised a series of treatment support strategies which includes Directly Observed Therapy (DOT), socioeconomic support, psychosocial and emotional support, education and counselling, early detection and effective management of adverse drug effects and non-adherent patients [3]. Among these strategies, DOT was recognized as the key element of DOTS and DOTS-Plus strategies [6], and has been recommended by WHO for all MDR-TB patients [3]. DOT was defined as an appointed agent providing the anti-TB drugs directly to the patient and watching as the patient swallows the medications [7]. A number of countries have included this strategy into their national MDR-TB control programs [5]. However in practice, many programs only provided DOT in the intensive phase or used patient self-administration during the DOTS period [4].

Providing DOT to MDR-TB patients for at least 20 months involves huge input from the program and patient sides. Therefore, it’s important to address the impact of implementing DOT among MDR-TB patients as it remains unclear. A recent meta-analysis of randomized controlled trials did not find any difference in treatment success rates between DOT and self-administration therapy (SAT) among drug-susceptible TB patients [8]. On the contrary, some observational studies suggested that DOT could leads to the best treatment results [9–11]. In addition, a previous systematic review indicated that DOT may reduce default rates of MDR-TB patients. To address both the scientific and health policy questions, we performed this systematic review and meta-analysis. The primary objective of this review is to determine the association between DOT and treatment success rates in MDR-TB patients. The secondary objective is to examine the impact of different DOT providers and DOT locations on successful treatment outcomes.

## Materials and Methods

This systematic review and meta-analysis was conducted according to the Preferred Reporting Items for Systematic Reviews and Meta-analyses (PRISMA) statement [12] (S1 Table).

### Search strategy

Two authors independently conducted the search work (YJ and XW). We systematically searched PubMed database, the ISI Web of Science, the Cochrane central register of controlled trials, EMBASE and CINAHL for articles published between January 1970 (approximately the date when MDR-TB was first reported) and December 2015. The following key words and medical subject heading terms were used as search terms: “MDRTB”; “MDR TB”; “MDR-TB”; “Multidrug-resistant Tuberculosis”; “directly observed therapy”; “DOT”; “DOTS”; “self-management”; “self-administration”; “default”; “interruption”; “management”; “adherence”, etc. Relevant articles listed in the reference lists of original articles identified from the electronic databases were hand-searched.

### Inclusion criteria

Articles were identified by screening titles first. The abstract was checked if the title of article was relevant. Then the full-text of articles with relevant abstracts would be reviewed. Results of eligible articles from the two reviewers were compared and disagreements were resolved by consensus. Articles were included if meet the following criteria: 1) Study design: the designs of studies were clinical trials, prospective or retrospective cohort studies, cross-sectional studies, case-control studies, or case series with at least 10 adult patients; 2) Participants: culturally confirmed MDR-TB patients with at least first-line drug susceptibility testing (DST) who received treatment with second-line anti-TB drugs; 3) Intervention: there was clear statement on using of full DOT, intensive phase DOT or SAT in the article. Studies introduced that patients were treated under DOT for a full course of therapy were classified as full DOT. Studies in which patients received DOT only in the intensive phase or during hospitalization in intensive phase were defined as intensive phase DOT. DOT was never provided or patients were treated with self-administration, was classified as SAT. Based on different DOT providers, we sub-divided studies used DOT into family-based DOT, healthcare provider based DOT (HCP-based DOT) and private DOT provider. Studies with DOT provided by patients’ family members were classified into family-based DOT, while studies with DOT provided by medical practitioners, nurses or public health practitioners were classified into HCP-based DOT. Studies with DOT provided by volunteers, community members who were not family members would be classified into private DOT provider. In terms of different DOT locations, studies were sub-divided into home-based DOT and health facility based DOT (HF-based DOT). Home-based DOT refers to delivering DOT to patients at home and HF-based DOT refers to providing DOT in TB clinics or comparable healthcare facilities. 4) Outcome: the final treatment outcomes have been reported, and the definitions and classifications of treatment outcomes were clear defined according to WHO guideline [13]. Cure and completed treatment were combined as treatment success. Articles were excluded if they were not published in English, or the reported studies were conducted exclusively among children less than 16 years of age, HIV patients or extensively drug-resistant tuberculosis (XDR-TB). We excluded XDR-TB patients where studies clearly stated XDR-TB patients in their subjects. However, early studies did not clearly report XDR-TB patients, thus we included all subjects but conducted a sensitivity analysis excluding studies in which patients were treated before 2000.

### Data extraction and analysis

An extraction form was designed and used to capture information from each eligible article. We recorded the detailed information of study locations, study years, study designs, number of participants, DOT types, DOT providers and locations, proportion of HIV co-infection, proportion of patients previously treated for TB, proportion of patients reported adverse effects, treatment regimens and treatment success rates. For duplicative studies involving overlapping authors, study period and location, we included the one with larger sample size and longer study period into this analysis. The excluded study was used to supplement some key information for which it may not be identified from the included one.

Comprehensive Meta-Analysis (version 2.0) was employed to conduct the analyses. Sample size and event rates was used as the data entry format. The meta-analysis was conducted by computing the pooled treatment success rate and 95% confidence interval (CI) using random effects model [14]. Heterogeneity was measured by Q and I^2^ statistic. Between-group difference was tested by using Q statistics and p-value. A p-value less than 0.05 indicated a significant difference. Also, non-overlapping CI demonstrated a significant difference between groups. I^2^ statistics and p values were used to explore heterogeneity between studies under each category [15]. An I^2^ value greater than 50% with p<0.05 indicated the existence of significant heterogeneity across studies. For studies using DOT (regardless of full or intensive phase DOT), we conducted subgroup analysis to examine if any particular type of DOT provider or DOT location was associated with a higher treatment success rate. Subgroup analyses were also carried out for all the included studies to determine the possible influence of other characteristics of studies (including sample size, study years, treatment regimens, proportion of HIV co-infection, proportion of previously treated TB cases, national income status classified according to report of International Monetary Fund [16], and the proportion of adverse effects being reported) on treatment success rates. A funnel plot was drawn together with Egger’s regression test to examine the potential existence of publication bias. We also performed sensitivity analysis to test if our main finding is robust by removing the studies in which the key information were failed to report, unclear or may cause publication bias.

## Results

**Fig 1:**
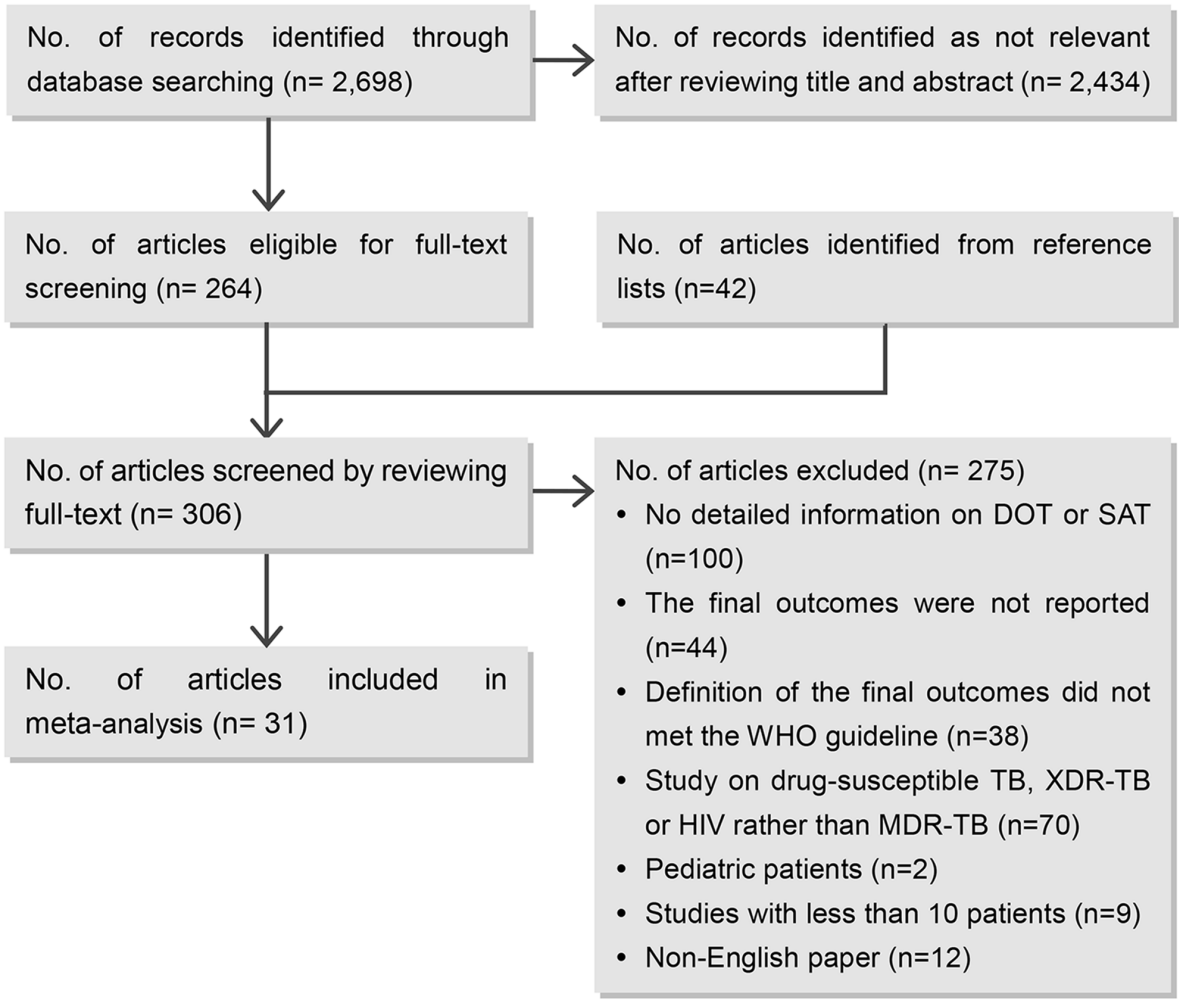
Summary of study selection process. No.: number; DOT: directly observed therapy; SAT: self-administration therapy; MDR-TB: multidrug-resistant tuberculosis; XDR-TB: extensively drug-resistant tuberculosis.

Originally, we identified 2,698 published articles from the electronic databases, of which 264 retained for full-text review after reviewing the titles and abstracts. Another 42 articles were identified from the reference lists of the relevance. Of the 306 articles which were reviewed for full-text, 275 were excluded as they failed to meet the inclusion criteria or duplicated with anther included study. A final of 31 articles [17–47] were eligible for this review (Fig 1).

### Study characteristics

We found no randomized trial on this study field. Majority of the articles were retrospective cohort studies (23 articles), seven were prospective cohort studies and one was cross-sectional study. Hoa’s article [21] was divided into two studies as a part of the participants were treated under full DOT and the others were treated with SAT. In total, 7,466 patients who were treated during 1983–2010 were included into our review, with the cohort size ranging from 25 to 1,209. The studies took place in 22 countries or regions, in which 13 were conducted in the advanced economic areas and the remaining 19 were from the developing economic areas. The proportion of HIV co-infection ranging from 0 to 12.4% in 19 of the 20 studies which reported the HIV results. The rest one conducted in South Africa had up to 51.8% of patients co-infected with HIV. Proportions of patients who were previously treated for TB ranged from 31.0% to 100% in 28 studies. Half of the studies reported adverse effects, and the proportions of adverse effects were lower than 30% in six studies, between 41.3% and 69.2% in four studies and more than 70% in the rest five. Patients were treated with individualized treatment regimen in 22 studies, with standardized treatment regimen in 9 studies, and with both treatment regimens in 1 study (S2 Table).

### Directly observation therapy (DOT)

In total, 19 studies implemented DOT for full course of treatment, four used DOT only in the intensive phase and the remaining nine used SAT. Of the 23 studies implementing DOT, 10 were HCP-based DOT, 2 were family-based DOT, 4 used private DOT providers, 5 used mixed providers and 2 did not report DOT provider. Among the five studies with mixed DOT providers, two used a mix of healthcare workers and family members, two used a mix of healthcare workers and private observers and one used all the three types of DOT providers. Regarding DOT location, 13 studies provided DOT in health facilities, 5 delivered DOT at home, 3 employed both locations, and 2 did not specify the type of DOT location. (S2 Table).

### Pooled estimate of treatment success rates for different DOT types

**Fig 2:**
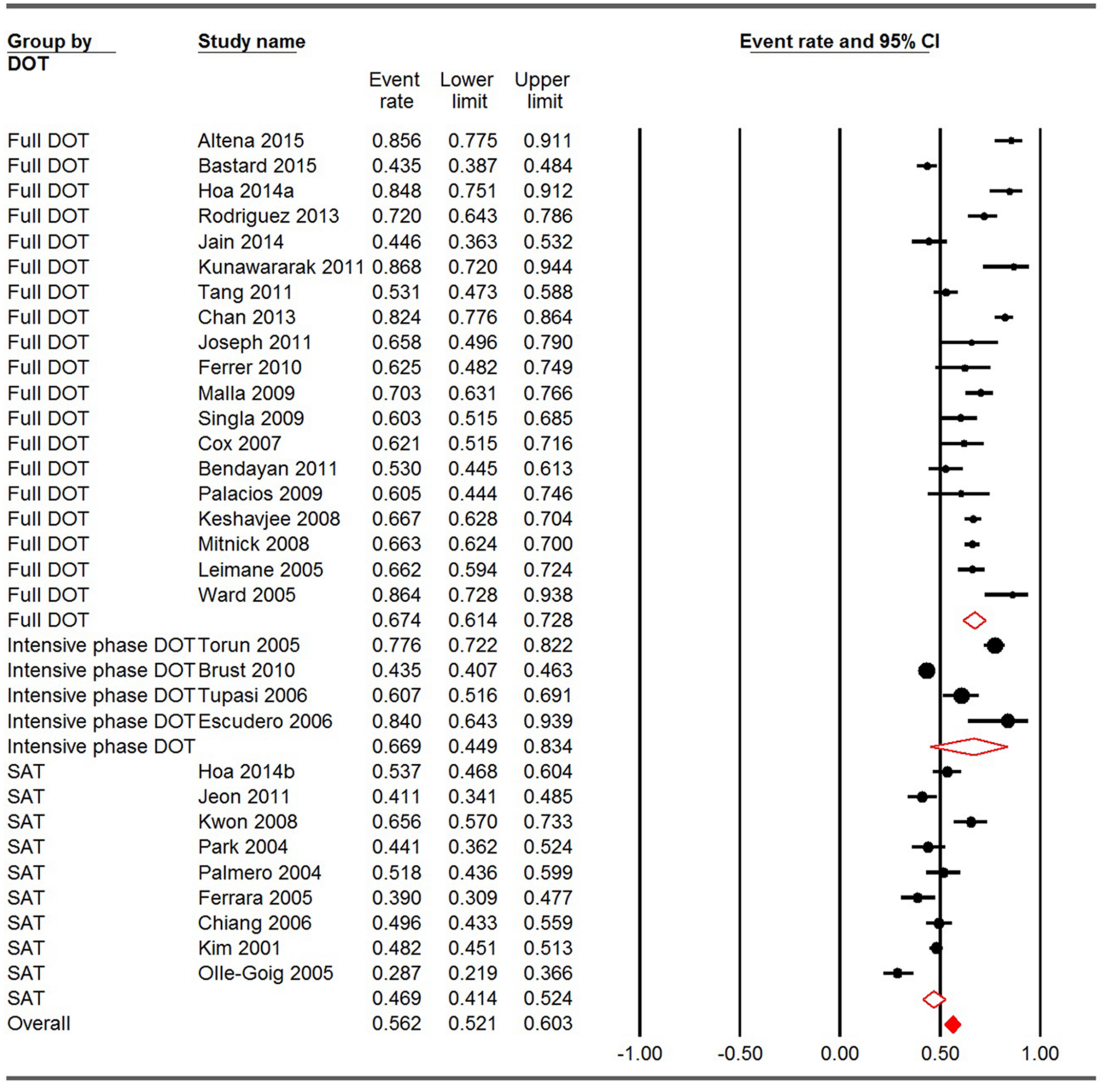
Meta-analysis of treatment success rates for studies using full DOT, intensive phase DOT and SAT. CI: confidence interval; DOT: directly observed therapy; SAT: self-administration therapy. I square for full DOT, incentive phase DOT and SAT were 91.323, 97.153 and 83.043, respectively. Q = 24.856, P<0.001. * The same study.

The overall treatment success rate for the 32 included studies (31 articles) was 56.2% (95% CI: 52.1–60.3%). The pooled treatment success rates for studies using full DOT, intensive phase DOT, and SAT were 67.4% (95%CI: 61.4–72.8%), 66.9% (95% CI: 44.9–83.4%), and 46.9% (95% CI: 41.4–52.4%), respectively. A statistical significance was found among these three groups (Q = 24.856, p<0.001). Compared with patients using SAT, those who were treated under full DOT had significantly higher treatment success rates (Fig 2).

### Pooled estimate of treatment success rates for different DOT providers and locations

**Fig 3:**
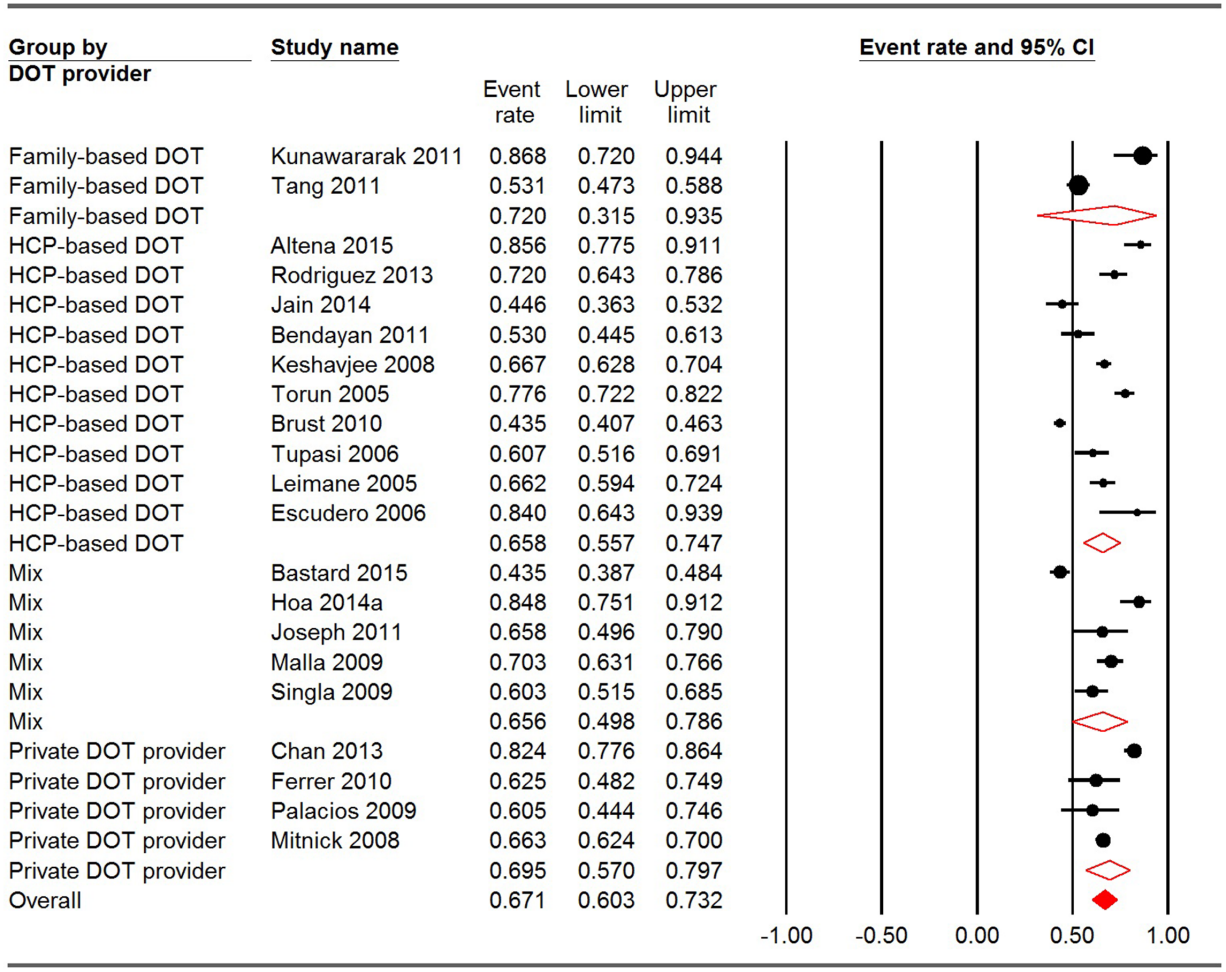
Meta-analysis of treatment success rates for studies using different DOT providers. CI: confidence interval; DOT: directly observed therapy; HCP: healthcare provider. I square for family-based DOT, HCP-based DOT, private DOT providers and mix were 92.125, 95.837, 88.967 and 93.660, respectively. Q = 0.343, P = 0.952.

**Fig 4:**
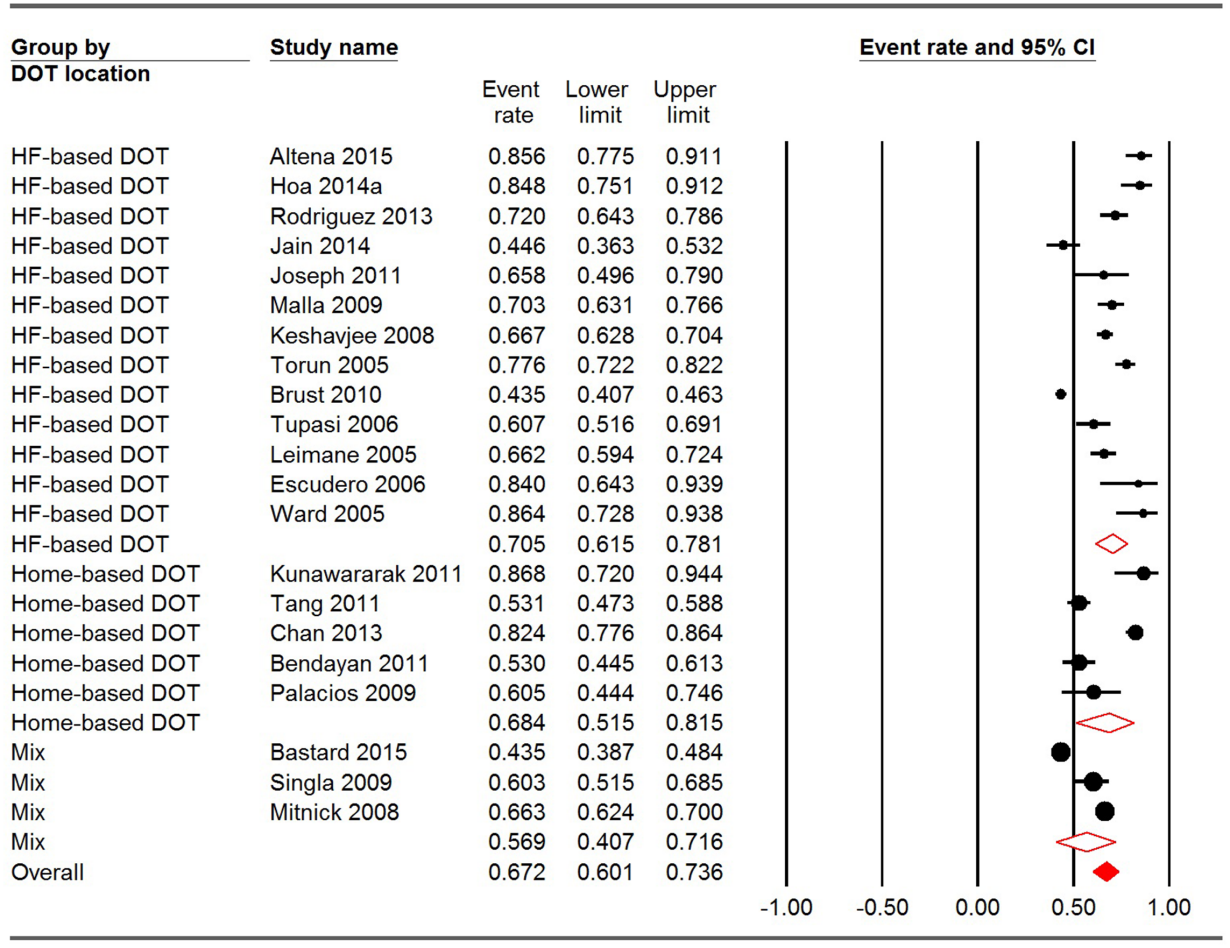
Meta-analysis of treatment success rates for studies using different DOT locations. CI: confidence interval; DOT: directly observed therapy; HF: health facility. I square for HF-based DOT, home-based DOT and mix were 95.412, 94.150 and 95.999, respectively. Q = 2.353, P = 0.308.

Among the 21 reports of DOT providers, the pooled treatment success rates for HCP-based DOT, family-based DOT, private DOT providers and a mix of them were 65.8% (95% CI: 55.7%-74.7%), 72.0% (95% CI: 31.5–93.5%), 69.5% (95% CI: 57.0–79.7%) and 65.6% (95% CI: 49.8–78.6%), respectively. Significant heterogeneity was observed within all the groups (P<0.001). The between-group variation, however, was not significant (Q = 0.343, P = 0.952) (Fig 3). Regarding the location of DOT, the pooled treatment success rates for HF-based DOT, home-based DOT and mixed places were 70.5% (95% CI: 61.5–78.1%), 68.4% (95% CI: 51.5–81.5%), and 56.9% (95% CI: 40.7–71.6%), respectively. Heterogeneity was also significant in all groups (p<0.001). Similar to DOT provider, between-group variation was not significant in terms of DOT location (Q = 2.353, p = 0.308) (Fig 4).

### Pooled estimate of treatment success rates in different sub-groups

Subgroup analyses were performed according to sample size, study years, treatment regimens, national income status, proportion of HIV co-infection, proportion of previously treated TB cases, and the proportion of adverse effects being reported. In Rodriguez’s study [22], 70% patients were treated with standardized treatment regimen and the others were treated with individualized treatment regimen. When analyzing the effect of treatment regimens on outcomes, we separated these two types of patients into corresponding groups. Thus, a total of 33 studies were analyzed by different treatment regimens. Studies with less than 100 sample size were more likely to have higher treatment success rates (74.5%, 95% CI: 64.9–82.2%) than studies with 100 to 200 sample size (55.6%, 95% CI: 47.1–63.9%). Compared with those who were treated across 1990s and 2000s (67.0%, 95% CI: 58.9–74.3%) and after 2000s (63.6%, 95% CI: 56.6–70.1%), patients who were treated before 2000s (43.7%, 95% CI: 36.9–50.9%) were more likely to report lower treatment success rates. The other factors did not demonstrate a significant contribution to any differences in treatment success rates (Table 1).

**Table 1:** Pooled treatment success rates by studies characteristics.

Studies characteristics	No. of studies	No. of patients included	Treatment success rate (95% CI)	I-square	Q-value	p-value
**Samples size**						
<100	8	397	74.5 (64.9–82.2)	72.278	8.804	0.0162
100–200	13	1790	55.6 (47.1–63.9)	91.878		
>200	11	5279	59.9 (52.0–67.3)	96.705		
**Study years**						
Before 2000s	5	1660	43.7 (36.9–50.9)	83.240	22.473	<0.001
Across 1990s and 2000s	9	1408	67.0 (58.9–74.3)	85.842		
In 2000s	18	4398	63.6 (56.6–70.1)	94.791		
**Treatment regimens**						
Standardized	10	2086	64.4 (54.4–73.3)	94.877	0.468	0.494
Individualized	23	5380	60.4 (54.4–66.2)	93.461		
**Economic areas**						
Advanced	13	3017	59.0 (50.8–66.8)	94.013	0.585	0.444
Developing	19	4449	63.0 (56.4–69.2)	94.011		
**HIV positive rate**						
0%	9	1099	66.8 (58.2–74.5)	85.838	4.826	0.090
>0%	11	3316	64.7 (55.7–72.7)	95.360		
Not reported	12	3051	54.5 (46.1–62.7)	94.575		
**Previous TB treatment**						
<90%	15	3201	61.8 (52.7–70.1)	95.035	0.181	0.913
≥90%	13	3328	60.6 (54.4–66.5)	90.667		
Not reported	4	937	63.8 (49.1–76.3)	94.699		
**Adverse effects**						
<70%	10	2638	61.3 (52.0–69.7)	94.289	1.901	0.387
≥70%	5	490	66.5 (59.2–73.2)	56.578		
Not reported	17	4338	59.6 (52.2–66.6)	95.020		

No.: number; CI: confidence interval; TB: tuberculosis.

### Publication bias and sensitivity analysis

**Fig 5:**
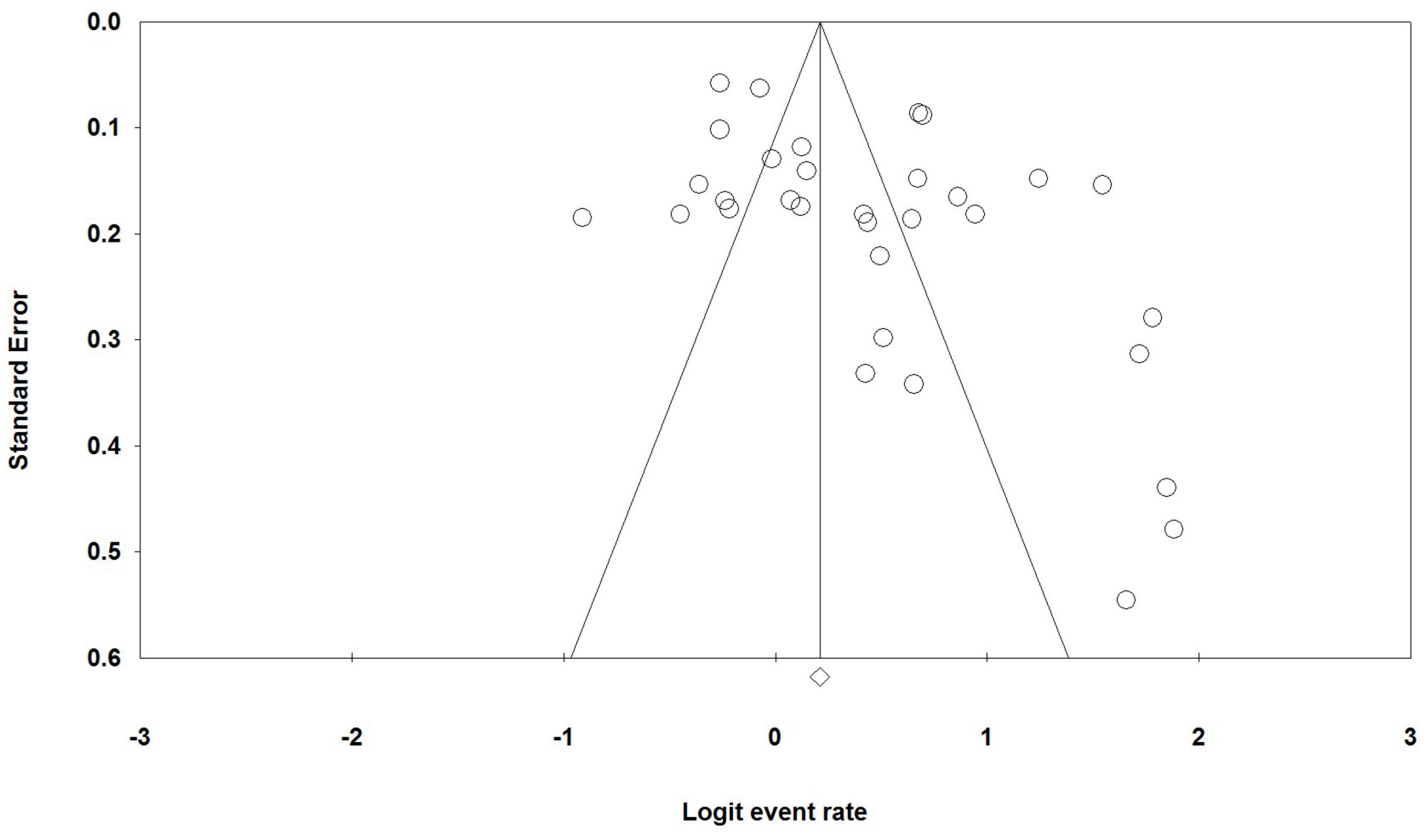
Funnel plot of standard error by logit event rate.

The publication bias exited as the funnel plot showed an asymmetrical pattern, and it can also be confirmed by the Egger’s regression test (p = 0.012) (Fig 5).

We performed three sensitivity analyses by removing three studies without reporting DOT provider or DOT location, five studies in which patients were treated before 2000s and one with unclear DOT type, respectively. The results showed that none of the sensitivity analysis changed the effect estimate (S3 Table).

## Discussion

All the studies included were observational studies. The results confirmed that full DOT associated with higher treatment success rates among MDR-TB patients. We found no evidence supporting that MDR-TB patients with DOT provided by healthcare workers had better treatment outcomes compared with those who had DOT provided by family members. Also, treatment success rates were not significantly different between studies using health facility based DOT and home-based DOT.

DOT was promoted by WHO and employed in many TB control programs to ensure patient long term adherence to the treatment. The rationale behind it was that the social interaction and peer pressure involved in DOT may motivate the patient to become more adherent to the prescribed treatment [8]. Although the effectiveness of DOT has been doubted for long, this study suggested that DOT did strengthen treatment outcomes among MDR-TB patients. On the other side, DOT caused some ethic and legal concerns [48, 49]. Some studies showed that frequent DOT may reveal patient status of having TB, which was a stigma in many cultures [50, 51]. To address this, WHO has advocated patient-centred care in its guideline [3]. However, this remains challenging of delivering DOT to MDR-TB patients with maximum convenience and respect.

In concordance with some previous studies [52–54], this review did not discover a better result among patients directly observed by healthcare providers compared with those observed by family members. That may due to the lengthy treatment duration for MDR-TB patients. Only 5–10% of drug susceptible TB patients were reported being fully DOT-ed in China [55, 56]. It is always challenging for anyone to provide daily DOT to patients for over 20 months. Poor implementation of DOT has been reported in many studies, though healthcare providers may be the named DOT providers [57, 58]. Studies found that family members, with more time to spend with the patients with caring minds, were more cost-effective in terms of time and resources compared to healthcare providers [59], and may improve MDR-TB treatment outcomes [60]. However, WHO did not recommend family members as a choice with the concern that they might not provide objective observation [3, 61]. In some countries, DOT was provided by well-trained private observers such as volunteers or dedicated DOT providers hired by government [62, 63]. These countries had observed relatively high MDR-TB treatment success rates. Under the condition of appropriate DOT training and effective supervision from healthcare system, private DOT providers may be feasible for rich counties with insufficient human resources.

Community-based treatment and care for MDR-TB is advocated by WHO [2]. Home-based DOT, is often more convenient to both patients and DOT providers [64]. This review found no significantly difference between home-based DOT and health facility based DOT regarding treatment success rates. But a recent observational study in South Africa revealed that MDR-TB patients under home-based care had higher treatment success rates than patients receiving care at health facilities [65], while similar results were found in China [60]. However, further studies, especially trials, need to be conducted to generate more robust study results.

Studies included in this review partially overlapped with those included in three previous reviews [4, 5, 66]. The participants in the previous ones were both MDR-TB and XDR-TB patients, while those in our review were only MDR-TB cases. Toczek’s review concluded that DOT may reduce default of MDR-TB, however, it failed to provide evidence on the relationship between DOT and treatment success rates, which may due to that some of the participants included in that review did not report final treatment outcomes. Neither of the other two used DOT as intervention in their study selection criteria.

Several limitations need to borne in mind. First, all the included studies were observational studies. Although the random controlled trials are desirable, there are practical limitations to conduct a trial with MDR-TB patients who need over 20-month treatment period. Thus, we conducted meta-analysis on observational studies to detect the effectiveness of intervention on this disease [67]. Second, some confounders that may also affect treatment success rates were not included, such as patient drug resistance patterns and other patient support strategies. Third, similar as the previous systematic reviews [4, 68] of observational studies, this review observed a significant heterogeneity in study characteristics. Fourth, we were not able to exclude XDR-TB patients in articles published early that did not report XDR in their subjects. We performed a sensitivity analysis by removing these articles while this did not change the conclusion. Another potential bias could be language selection bias, as we had only included articles published in English. Moreover, incomplete data regarding DOT providers, DOT locations, HIV prevalence, proportions of previously treated cases, and patient socio-economic characteristics were reported in several of our included studies, which may prevent us having a robust pooled treatment success rate.

## Conclusions

We found that MDR-TB patients having full DOT were more likely to achieve higher treatment success rates. Further studies are needed to compare the effectiveness of different DOT providers and locations regarding treatment success rates.

## Supporting Information

S1 Table PRISMA Checklist for the systematic review. (DOC)

S2 Table Characteristics of studies that included in the systematic review. (DOCX)

S3 Table Sensitivity analysis. (DOCX)
