# Supplementary material for: Association between Directly Observed Therapy and Treatment Outcomes in Multidrug-Resistant Tuberculosis: A Systematic Review and Meta-Analysis
Source: PLoS One. 2016 Mar 1;11(3):e0150511. doi: 10.1371/journal.pone.0150511 (PMC4773051; doi:10.1371/journal.pone.0150511)
Supplement: S2 Table — (DOCX) [file pone.0150511.s002.docx]

S2 table. Characteristics of studies that included in the systematic review

| Study name | Study location | Study year | Study design | No. of patients Included | HIV positive, no. (%) | Previously treated, no. (%) | Treatment regimen | Adverse effects reported no. (%) | Treatment success rate, no. (%) | DOT | DOT provider | DOT location |
| --- | --- | --- | --- | --- | --- | --- | --- | --- | --- | --- | --- | --- |
| Altena 2015 | Netherlands | 2000-2009 | RC | 104 | 14/113 (12.4) | 35/113 (31.0) | Individualized | 43 (41.3) | 89 (85.6) | Full DOT | Nurse of TB centre | TB centre |
| Bastard 2015 | U.S. | 2002-2010 | RC | 393 | NR | 304 (77.4) | Individualized | NR | 171 (43.5) | Full DOT | Mix (health worker or community member) | Mix (health facility or home) |
| Hoa 2014a^a^ | Vietnam (GLC) | 2010 | RC | 79 | 3 (3.8) | 79 (100) | Standardized | NR | 67 (84.8) | Full DOT | Mix (health worker in the district TB unit and family member) | District TB unit (or commune heath post) |
| Hoa 2014b^a^ | Vietnam (Non-GLC) | 2010 | RC | 203 | 2 (1) | 200 (98.5) | Individualized | NR | 109 (53.7) | SAT |  |  |
| Rodriguez 2013 | The Dominican Republic | 2006-2008 | RC | 150 | NR | NR | Standardized (105 ); individualized (45) | NR | Total: 108 (72.0)  Standardized: 78 (74.3)  Individualized: 30 (66.7) | Full DOT | Nurse | Community health centre |
| Jain 2014 | India | 2009 | PC | 130 | NR | 130 (100) | Standardized | 35 (26) | 58 (44.6) | Full DOT | 80% health care workers and 20% medical doctors | Community health centre |
| Kunawararak 2011 | Thailand | 2008-2009 | CS | 38 | 3 (7.9) | 25 (65.8) | Standardized | NR | 33 (86.8) | Full DOT | Family member | Home |
| Tang 2011 | China | 2007-2009 | RC | 286 | 0 | NR | Individualized | NR | 152 (53.1) | Full DOT | Family member | Home |
| Chan, 2013^b^ | Taiwan | 2007-2008 | RC | 290 | NR | 172 (59.3) | Individualized | NR | 239 (82.4) | Full DOT | Private provider hired by health centres | Home |
| Joseph 2011 | India | 2006-2007 | PC | 38 | 0 | 38 (100) | Standardized | 33 (86.8) | 25 (65.8) | Full DOT | Mix (health care providers, private medical practitioners, or family members) | Community health centre |
| Ferrer 2010 | U.S. | 1994-2007 | RC | 48 | 0 | 37 (77.1) | Individualized | NR | 30 (62.5) | Full DOT | Social workers | NR |
| Malla 2009 | Nepal | 2005-2006 | RC | 175 | NR | 163 (93.1) | Standardized | NR | 123 (70.3) | Full DOT | Mix (nurses and community members) | Clinic |
| Singla 2009 | India | 2002-2006 | RC | 126 | 0 | 126 (100) | Standardized | 73 (57.9) | 76 (60.3) | Full DOT | Mix (health care workers and family members) | Mix (health care centre and home) |
| Cox 2007 | Uzbekistan | 2003-2005 | PC | 87 | NA | 87 (100) | Individualized | 67 (77.0) | 54 (62.1) | Full DOT | NR | NR |
| Bendayan 2011 | Israel | 2000-2005 | PC | 132 | 8 (6.1) | 77 (58.3) | Individualized | 38 (28.8) | 70 (53.0) | Full DOT | Nurse | Home |
| Palacios 2009 | Peru | 1996-2005 | RC | 38 | 3 (7.9) | 34 (89.5) | Individualized | NR | 23 (60.5) | Full DOT | DOT volunteers | Home |
| Jeon 2011 | Korea | 2004 | RC | 175^c^ | NA | 141 (80.6) | Individualized | NR | 72 (41.1) | SAT |  |  |
| Keshavjee 2008 | Russia | 2000-2004 | RC | 579^c^ | 5 (0.9) | 579 (100) | Individualized | 388 (67.0) | 386 (66.7) | Full DOT | Physicians | Clinic |
| Kwon 2008 | Korea | 1995-2004 | RC | 128^c^ | 0 | 113 (88.3) | Individualized | NR | 84 (65.6) | SAT |  |  |
| Torun 2005 | Turkey | 1992-2004 | RC | 263 | 0 | NR | Individualized | 182 (69.2) | 204 (77.6) | IP DOT | nurse | Hospital |
| Brust 2010 | South Africa | 2000-2003 | RC | 1209 | 362/699 (51.8) | 959/1191 (80.5) | Standardized | NR | 526 (43.5) | IP DOT | nurse | Hospital |
| Mitnick 2008 | Peru | 1999-2002 | RC | 603^c^ | 9 (1.5) | 602 (99.8) | Individualized | NR | 400 (66.3) | Full DOT | DOT volunteers | Mix (health care centre and home) |
| Tupasi 2006 | Philippines | 1999-2002 | PC | 117 | NR | 112 (95.7) | Individualized | 112 (95.7) | 71 (60.7) | IP DOT | Staff of medical center | Clinic of hospital |
| Leimane 2005 | Latvia | 2000 | RC | 204 | 1 (0.5) | 149 (73.0) | Individualized | 176 (86.3) | 135 (66.2) | Full DOT | Nurse | Clinics |
| Escudero 2006 | Spain | 1998-2000 | PC | 25 | 0 | 22 (88.0) | Individualized | 7 (28.0) | 21 (84.0) | IP DOT | Nurse | Hospital |
| Park 2004 | Korea | 1998-2000 | RC | 142 | NR | 142 (100) | Standardized | NR | 63 (44.1) | SAT |  |  |
| Ward 2005 | Vietnam | 1989-2000 | RC | 44 | 0 | 34 (77.3) | Standardized | 31 (70.5) | 38 (86.4) | Full DOT | NR | Clinic |
| Palmero 2004 | Argentina | 1996-1999 | PC | 141 | 0 | 91 (64.5) | Individualized | 33 (23.4) | 73 (51.8) | SAT |  |  |
| Ferrara 2005 | Italy | 1995-1999 | RC | 127 | 15 (11.8) | 75 (59.1) | Individualized | 22 (17.3) | 49 (39.0) | SAT^e^ |  |  |
| Chiang 2006 | Taiwan | 1992-1996 | RC | 238 ^d^ | NR | NR | Individualized | NR | 118 (49.6) | SAT |  |  |
| Kim 2001 | Korea | 1988-1996 | RC | 1011 | NA | 1011 (100) | Individualized | 90 (8.9) | 487 (48.2) | SAT |  |  |
| Olle-Goig 2005 | Bolivia | 1983-1993 | RC | 143 | NR | 103 (72.0) | Individualized | NR | 41 (28.7) | SAT |  |  |

no.: number; RC: retrospective cohort study; PC: prospective cohort study; CS: cross-sectional study; NR: not reported; NA: not available; GLC: Green Light Committee; DOT: directly observed therapy; SAT: self-administration therapy; IP: incentive phase.

^a^ The same study.

^b^ Patients who were not treated within the program of Taiwan MDR-TB Consortiums (TMTC) were excluded as no DOT related information was provided.

^c^ Patients who were XDR-TB were excluded.

^d^ Patients who did not treated with second-line anti-TB drug were excluded.

^e^ Only 38% of patients received DOT during hospitalization.
