# Supplementary material for: Association between Directly Observed Therapy and Treatment Outcomes in Multidrug-Resistant Tuberculosis: A Systematic Review and Meta-Analysis
Source: PLoS One. 2016 Mar 1;11(3):e0150511. doi: 10.1371/journal.pone.0150511 (PMC4773051; doi:10.1371/journal.pone.0150511)
Supplement: S3 Table — (DOCX) [file pone.0150511.s003.docx]

S3 table. Sensitivity analysis

| Variables | No. of studies | Pooled treatment success rate (95% CI) | Q-value | p-value |
| --- | --- | --- | --- | --- |
| Removal of studies without reporting DOT provider or DOT location | 29 |  |  |  |
| Full DOT | 16 | 66.9 (60.4-72.8) | 21.636 | ＜0.001 |
| Intensive phase DOT | 4 | 66.9 (44.9-83.4) |  |  |
| SAT | 9 | 46.9 (41.4-52.4) |  |  |
| Removal of the early studies (study year was before 2000s) | 27 |  |  |  |
| Full DOT | 19 | 67.4 (61.4-72.8) | 7.633 | 0.022 |
| Intensive phase DOT | 4 | 66.9 (44.9-83.4) |  |  |
| SAT | 4 | 51.1 (40.8-61.2) |  |  |
| Removal of the study with uncertain DOT classification | 31 |  |  |  |
| Full DOT | 19 | 67.4 (61.4-72.8) | 21.550 | ＜0.001 |
| Intensive phase DOT | 4 | 66.9 (44.9-83.4) |  |  |
| SAT | 8 | 47.8 (42.0-53.7) |  |  |

No.: number; DOT: directly observed therapy; SAT: self-administration therapy; CI: confidence interval.
